# An analysis of pre-season risk factors for low back injury in high-school baseball pitchers: a prospective study

**DOI:** 10.1038/s41598-021-90988-9

**Published:** 2021-06-01

**Authors:** Kurumi Nakase, Hitoshi Shitara, Tsuyoshi Tajika, Takuro Kuboi, Tsuyoshi Ichinose, Tsuyoshi Sasaki, Noritaka Hamano, Fumitaka Endo, Masataka Kamiyama, Ryosuke Miyamoto, Atsushi Yamamoto, Tsutomu Kobayashi, Kenji Takagishi, Hirotaka Chikuda

**Affiliations:** Department of Orthopaedic Surgery, Gunma University Graduate School of Medicine, 3-39-22, Showa, Maebashi, Gunma 371-8511 Japan

**Keywords:** Anatomy, Medical research, Risk factors

## Abstract

Pitching motion requires whole-body coordination; therefore, poor control of the lower extremities, pelvis and trunk may cause shoulder and elbow injuries. However, few studies have described the relationship between the shoulder joint function and low back injury in high-school baseball pitchers. A total of 128 healthy high school pitchers underwent pre-season medical checkups, where their shoulder range of motion and shoulder strength were measured. The participants completed a self-recorded daily questionnaire regarding the presence of low back pain. Pitchers were divided into injured and non-injured groups. Low back injury was observed in 13 participants (13.4%). In the injured group, horizontal adduction on the dominant shoulder was significantly less than in the non-injured group. A logistic regression analysis showed that horizontal adduction on the dominant side was a significant independent risk factor for low back injury during the season. It is important to recognize that restriction of the shoulder function not only causes shoulder and elbow injuries but can also risk low back injury.

## Introduction

Pitching requires whole-body coordination, and poor control of the lower extremities, pelvis and trunk may cause shoulder and elbow injuries. In high-school baseball pitchers, limitations in dominant shoulder internal rotation measured during pre-season medical checkups are a risk factor for the development of shoulder and elbow injuries during the season^1^. The lumbar spine is reported to be essential in the kinematics of pitching, since the energy transfers from the lower to upper of the body during pitching^2^. In previous studies, the low back injury rates have ranged from 8.3% to 12% among professional and college baseball players^3,4^, and the prevalence of chronic low back pain ranges from 1 to 40% among baseball players of all experience levels^5,6^.

Among high-school baseball players, 49.7% of players experienced low back pain during 1-year follow-up^7^. In addition, hamstring tightness on the non-throwing arm side was identified as a potential risk factor for low back pain in high-school baseball players^7^. Although a potential risk factor for low back pain has been identified in the lower extremities, no study has been conducted for the upper extremities. Furthermore, the mechanisms underlying the occurrence of low back pain may differ between field players and pitchers because pitchers mainly perform pitching and field players mainly perform batting and defense practice in daily practice.

Because the risk factors for low back pain in the upper extremities in high-school baseball pitchers have not been investigated, we investigated the relationship between the occurrence of low back pain and deficits of the shoulder functions, which are known risk factors for baseball-related upper extremity injury. We hypothesized that deficiencies in the shoulder function at pre-season medical checkups might cause low back injury, as when the upper extremities cannot provide normal pitching power, trunk flexion and rotation might exert excessive motion to compensate.

Therefore, the present study prospectively explored the risk factors associated with the shoulder function for low back injury occurring during the season among high-school baseball pitchers based on pre-season medical checkups.

## Methods

The institutional review board of Gunma University Hospital (Identification number 1003) approved this study. All methods were carried out in accordance with relevant guidelines and regulations. Written informed consent was obtained from the parents of the participants.

In this prospective study, we examined 128 high school male baseball pitchers who were 15 to 17 years of age. The inclusion criteria^8^ included players who had: (1) participated in pre-season medical checkups in 2018; (2) participated in preseason practice as an active player; (3) had no restrictions in baseball activities, including throwing, running, and batting; and (4) had completed a daily questionnaire about the presence of low back pain, which was collected every month throughout the season. The exclusion criteria^8^ were: (1) prior injury to the throwing arm or low back and (2) an inability to play baseball due to foot, ankle, knee, hip, spine, shoulder, or elbow injuries.

### Pre-season medical checkups

As previously reported^1,9^, pre-season medical checkups were performed as baseline medical examinations. To avoid confirmation bias, participants’ hand dominance was not announced to examiners. The participants completed a demographic questionnaire that included their height, weight, baseball experience, and hand dominance.

During the physical, an orthopedic surgeon measured their shoulder range of motion; shoulder external rotation, internal rotation, and horizontal adduction and shoulder strength; and abduction, external rotation, and internal rotation.

### Shoulder range of motion and strength measurements

The intra-rater and inter-rater reliability of digital protractors have been established in the literature^1^. According to previously reported methods^1,9–15^, a certified orthopedic surgeon assessed the passive shoulder range of motion of 90° abducted external and internal rotation (ABER, ABIR) and horizontal adduction on both the dominant and non-dominant shoulders using a digital protractor with a bubble level indicator (iGaging, Los Angeles, CA, USA). The participant was placed in the supine position with legs straight on the examination table, with the shoulder abducted to 90° and elbow flexed to 90º. A small, rolled towel was placed under the elbow to keep the humerus in the right position. The scapula was stabilized posteriorly against the examination table by applying pressure to the coracoid process using the thumb and thenar eminence. The humerus was passively rotated externally and internally until an end feel was obtained and the scapula began to move. The axis of the digital protractor was placed on the olecranon process of the elbow with the stationary arm aligned vertically, and the moving arm was aligned with the forearm. The total arc was calculated for the dominant shoulder by adding the ABER and ABIR.

To measure the shoulder horizontal adduction, the participant was placed in the supine position on the examination table with the scapula stabilized by the examiner’s pressure on the lateral border of the scapula with the thenar eminence. The test shoulder and elbow were positioned in 90° of both flexion and abduction. The examiner’s opposite hand then held the participant’s forearm, and the humerus was passively moved into horizontal adduction until an end feel was obtained and the lateral border of the scapula began to move. The axis of the digital protractor was placed at the estimated center of the glenohumeral joint with the stationary arm perpendicular to the horizontal plane, and the moving arm was aligned with the humerus. All shoulder range of motion measurements were performed once by two examiners with one examiner providing stabilization force to maintain the shoulder position while the other obtained the range of motion. The range of motion was measured before the muscle strength.

### Shoulder strength measurements

The intra-rater and inter-rater reliability of the shoulder strength measurements by hand-held dynamometers have been established in a previous study^1^. In accordance with previous studies^1,9,16^, using a PowerTrack II Commander hand-held dynamometer (J-Tech Medical, Salt Lake City, UT, USA), a certified orthopedic surgeon measured the strength of the supraspinatus three times in the seated position (SS), prone external rotation (PER) and prone internal rotation (PIR) in both the dominant and non-dominant shoulders. When the SS strength was measured, the participant sat on the examination table with his back against the wall. The humerus was abducted to 90° and then horizontally adducted to 45° with the forearm neutral. The examiner placed the dynamometer 5 cm proximal to the proximal wrist extension crease, and the participant raised his arm perpendicular to the floor with maximum effort. The PER and PIR strength were measured in the prone position with the shoulder abducted to 90º and the elbow flexed to 90º. The examiner stabilized the humerus and set the arm in 0º of rotation, and then the participant rotated his arm externally and internally with maximum effort against the dynamometer. When the PIR strength was measured, the dynamometer was placed 5 cm proximal to the proximal wrist flexion crease, and when the PER strength was measured, the dynamometer was placed at the dorsal side of the forearm, opposite to the proximal wrist flexion crease. The median value of the three repetition trials was recorded and subsequently analyzed.

### Low back injury

In this study, “low back injury” was defined as any condition that resulted in the pitcher being considered disabled for eight days or longer^1^. Other injuries caused by other mechanisms, such as being hit by a ball, colliding with other players, or suffering trauma from falls, were excluded. To avoid recall bias, participants were instructed to complete a self-recorded questionnaire every day regarding the presence of low back pain during pitching, limitations to pitching caused by low back pain, and the presence of other injuries.

### Statistical analyses

Statistical analyses were performed using the SAS 9.4 software program (SAS Institute Inc., Cary, NC, USA). All tests were two-sided with a *P* = 0.05 significance level. Depending on the presence of low back injuries, the participants were divided into injured and non-injured groups. Categorical data were reported as the frequency (%), and group differences were evaluated using the chi-square test. Continuous data were reported as mean with standard deviation (SD), and group differences were evaluated using the Mann–Whitney U-test. After adjusting for significant variables identified in univariate analyses, the logistic regression analysis was performed to calculate odds ratios (ORs) and 95% confidence intervals (CIs) to identify the risk factors for low back pain.

To determine the sample size for this study, a prior statistical power analysis for a logistic regression analysis was performed. This analysis indicated that a total of 70 participants would be needed, depending on a statistical power of 80% at an α level of 0.05 (assumptive incidence rate = 20%, OR ratio = 2.5)^17^.

## Results

**Figure 1:**
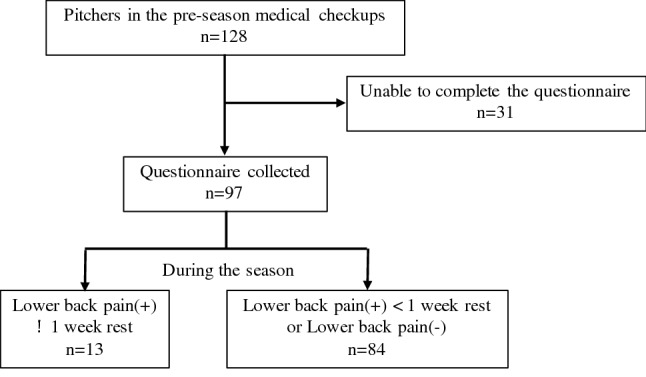
A flow chart to identify the pitchers included in this study.

A total of 128 high-school baseball pitchers participated in pre-season medical checkups. We were able to collect daily questionnaires monthly during the season from 97 pitchers, with 31 pitchers not answering the questionnaires. We therefore enrolled these 97 pitchers in the final analysis. The response rate for daily questionnaires among the 97 pitchers was 100%. Low back injury was observed in 13 participants (13.4%) during the season (Fig. 1).

In the preseason baseline assessment, there were no significant differences between non-injured and injured group in height (*P* = 0.250; 173.5 ± 7.2 and 170.7 ± 3.4 cm, respectively) and baseball experience (*P* = 0.210; 8.4 ± 1.7 and 9.2 ± 1.7 years, respectively).

The dominant shoulder horizonal adduction was significantly lower in the injured group than in the non-injured group (*P* = 0.013). No significant differences were observed between the injured and non-injured groups with respect to horizontal adduction on the non-dominant side, ABER, ABIR (Table 1). There were also no significant differences in shoulder strength between the groups (Table 2).

**Table 1:** Shoulder range of motion.

	Non-injured n = 84		Injured n = 13		*P* value
	Mean	SD	Mean	SD	
*Dominant side (°)*					
External rotation (90° abduction)	108.1	10.6	106.4	8.1	0.388
Internal rotation (90° abduction)	37.8	10.7	40.9	10.1	0.433
Total arc	145.9	14.8	147.3	14.1	0.691
Horizontal adduction	9.3	9.7	2.5	12.7	0.013*
*Non-dominant side (°)*					
External rotation (90° abduction)	100.9	9.6	97.1	4.0	0.113
Internal rotation (90° abduction)	47.1	11.4	44.7	9.1	0.187
Total arc	148.0	13.7	141.8	9.0	0.110
Horizontal adduction	21.9	10.7	18.6	7.3	0.388

SD, standard deviation.

*P < 0.05.

**Table 2:** Shoulder strength.

	Non-injured n = 84		Injured n = 13		*P* value
	Mean	SD	Mean	SD	
*Dominant side (kg)*					
Seated supraspinatus	9.4	1.8	9.7	1.8	0.437
Prone external rotation	13.8	3.3	14.1	4.6	0.945
Prone internal rotation	17.3	4.2	17.9	5.1	0.865
*Non-dominant side (kg)*					
Seated supraspinatus	9.3	1.8	9.5	1.8	0.193
Prone external rotation	14.3	3.6	14.8	3.2	0.141
Prone internal rotation	17.4	4.2	17.4	4.8	0.751

SD, standard deviation.

*P < 0.05.

A logistic regression analysis showed that horizontal adduction on the dominant side was a significant independent risk factor for low back injury during the season (*P* = 0.010, OR 0.92, 95% CI 0.87–0.98).

## Discussion

The most important finding of this study was that the range of motion deficit of horizontal adduction on the dominant shoulder was an independent risk factor for baseball-related low back injury in high-school baseball pitchers. Furthermore, we showed that if horizontal adduction on the dominant side were improved by 13.8°, which was the difference in the range of motion between the injured and non-injured group, the injury risk would be reduced by 68% (calculated OR: 0.32). These findings may help prevent low back injury as well as shoulder and elbow injury.

The prevalence of low back injury in this study of high-school baseball pitchers was 13.4%, which was similar to that in a previous study^5,6^. However, Kato et al. showed that 49.7% of high-school baseball players suffered from low back pain during 1-year follow-up^7^. This marked difference in the occurrence rate of low back pain between the present and Kato’s study might be due to the target populations. Of note, the shoulder range of motion and strength in this study were similar to those found in previous checkups in our facility^1^.

### The relationship between lower limb, trunk and upper limb injuries

In pitching kinematics, the trunk begins to rotate toward to home plate in the late cocking phase and flexes in the acceleration phase^2^, while horizontal adduction of the shoulder joint is observed from the acceleration through the deceleration phase^18^.

Previous studies have investigated the association of deficits in the trunk and lower extremities with shoulder and elbow pain and injury in adolescent baseball players. In those studies, deficiencies in the hip range of motion and the existence of low back pain were independent risk factors for shoulder and elbow injuries. Sekiguchi et al. investigated 1582 young baseball players (6–15 years old) using a questionnaire concerning their shoulder, elbow, low back, and knee pain. The results showed that the presence of low back and knee pain was significantly associated with the prevalence of shoulder and/or elbow pain in both pitchers and non-pitchers^19^. They also demonstrated that restriction of hip rotation in the stride leg was associated with shoulder and elbow pain in young baseball players (9–12 years old)^20^. A total of 177 participants (44 pitchers, 133 non-pitchers) were included in their analysis, and 9% (5 pitchers, 11 non-pitchers) developed shoulder and elbow pain during a 3-day-tournament. They also showed significant restriction of the hip internal rotation of the stride leg, which had been measured just before the tournament^20^.

These previous studies indicated that either hip or trunk function deficiency would lead to shoulder or elbow pain in young baseball pitchers or players^19,20^. In other words, efficient throwing without shoulder or elbow injury depends on a player’s hip or trunk function, which play an important role in pitching kinematics. Thus, inappropriate flexibility at any point in this pitching motion sequence can lead to an increased risk of throwing-arm injury. However, these previous studies were either cross-sectional or case–control studies, so whether the hip range of motion limitations occurred before or after shoulder and/or elbow pain has been unclear. In addition, the participants in those studies were younger than those in our own.

### Shoulder range of motion and injuries

Shanley et al. investigated 246 high-school softball and baseball players (51 pitchers, 195 non-pitchers), and 27 shoulder and elbow injuries (12 pitchers, 15 non-pitchers) were observed during the season. Furthermore, the horizontal adduction on the dominant side was significantly lower in the injured players’ dominant shoulder than in the uninjured players’ dominant shoulder^11^.

To our knowledge, this is the first prospective study to report the risk of low back injury based on shoulder range of motion data measured in pre-season medical checkups before the development of low back injury among high-school baseball pitchers. This study showed that the injured group had a significantly lower horizontal adduction in the dominant shoulder than the uninjured group. Based on the present and previous findings, it is plausible that a decreased horizontal adduction on the dominant may lead to excessive trunk rotation, and a lack of horizontal adduction may cause the pitcher to be unable to transfer energy from the legs to the arm efficiently, leading to excessive use of the trunk and thus causing low back injury. Further research is necessary to determine whether or not players with poor horizontal adduction of the shoulder range of motion actually use their low back muscles more than others when pitching.

### Limitations

Several limitations associated with this study should be acknowledged. Our intention for this study was to evaluate the relationship between the shoulder function and low back injury, so factors other than horizontal adduction may have affected the results. First, we did not consider other external load factors, such as batting, total number of pitches, or number of innings pitched, which might have affected the lower back condition. Second, we did not evaluate the trunk condition in detail at the pre-season medical checkup. However, we believe that we did not enroll participants who had severe low back conditions because we conducted a screening test by asking if participants had low back problems that affected their pitching performance. Third, we did not consider repeated low back injuries within a pitcher in this study. The investigation of the characteristics of pitchers who experienced repeated low back injuries might help prevent severe low back injuries. Fourth, we did not collect data on the severity or precise location of low back pain. Finally, the sample size was small because the incidence of low back injury is low among high-school baseball pitchers. However, the number of participants matched the condition that the prior power analysis required (total 70 participants). Further studies are needed to resolve the above issues.

## Conclusion

In summary, we performed a prospective analysis using the data from pre-season medical checkups for high-school baseball pitchers to elucidate the relationship between the shoulder joint function and low back injury. A range of motion deficit of horizontal adduction on the dominant shoulder was a significant independent risk factor for baseball-related low back injury in high-school baseball pitchers. Furthermore, we found that if horizontal adduction on the dominant side were improved by 13.8°, which was the difference in the range of motion between the injured and non-injured group, the injury risk would be reduced by 68%. These findings may help prevent low back injury as well as shoulder and elbow injury.
